# Machine learning to dissect perturbations in complex cellular systems

**DOI:** 10.1016/j.csbj.2025.02.028

**Published:** 2025-02-26

**Authors:** Pablo Monfort-Lanzas, Katja Rungger, Leonie Madersbacher, Hubert Hackl

**Affiliations:** a Institute of Bioinformatics, Biocenter, Medical University of Innsbruck, Austria; b Institute of Medical Biochemistry, Biocenter, Medical University of Innsbruck, Austria

**Keywords:** Artificial intelligence, Machine learning, Perturbation, Dose response, CRISPR-Cas9 screening, Single cell RNA sequencing, Spatial transcriptomics

## Abstract

Understanding the responses of biological systems to various perturbations, such as genetic, chemical, or environmental challenges, is essential for reconstructing causal network models. Emerging single-cell technologies have become instrumental in elucidating cell states and phenotypes and they have been used in combination with genetic screening. Recent advances in machine learning and artificial intelligence architectures have stimulated the development of computational tools for modeling perturbations and the response to compounds. This study outlined core principles underpinning perturbation analysis and discussed the methodologies and analytical frameworks used to decode drug and genetic perturbation responses, complex multicellular interactions, and network dynamics. The current tools used for various applications were overviewed. These developments hold great promise for improving drug development and personalized medicine. Foundation models and perturbation cell and tissue atlases offer immense potential for advancing our understanding of cellular behavior and disease mechanisms.

## Graphical Abstract

**Figure:**
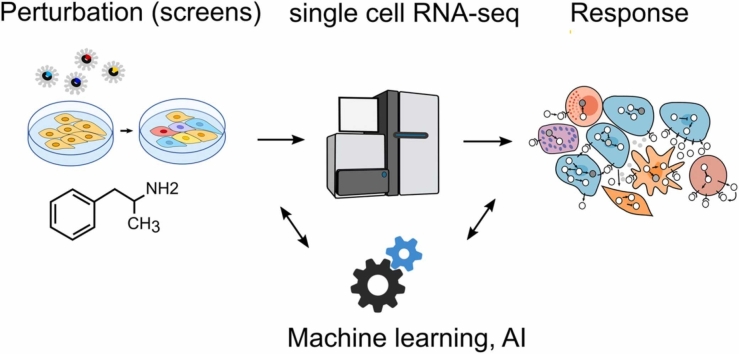

## 1 Introduction

Complex biological systems are organized hierarchically, from the molecular level to tissues or heterogeneous cellular structures. Emerging single-cell technologies have facilitated the elucidation of cell states and phenotypes. In addition to methods analyzing the transcriptomics layer, such as single-cell RNA sequencing (scRNA-seq) and spatial transcriptomics, technologies for the comprehensive analyses of proteins, metabolites, multiomics integration, and imaging have been developed. However, dynamic biological systems are organized through a complex network of interconnected pathways that maintain cellular homeostasis. To obtain a mechanistic understanding of system behavior, termed systems identification in technical system analysis, it is necessary to perturb the system. Understanding the responses of biological systems to various perturbations, such as genetic, chemical, or environmental challenges, is essential for reconstructing causal network models. This can be achieved through the rational manipulation of cells, which is crucial in modern biomedical research [1], [2].

This development has significantly influenced precision medicine, in which chemical and therapeutic interventions are studied in disease models such as organoids. Perturbation modeling has become critical for developing personalized treatments, controlling disease progression, and identifying therapeutic targets. It is also essential for precise engineering of cells for regenerative therapies. Experimental methods such as CRISPR or RNAi technologies permit the genetic manipulation of individual genes and enable the performance of pooled genetic screens across many genes in combination with readout from scRNA-seq (Perturb-Seq), providing ideal approaches to address the perturbation response in various applications [3].

Given the high number of potential molecular changes and their often non-linear responses, perturbation analysis possesses high combinatorial complexity. Different modeling approaches capture the responses of biological systems to perturbations. Network analyses map key interactions and regulatory patterns [4], [5], [6]. Dynamic logic models [7], [8], [9] describe system-wide changes without requiring detailed kinetic data. Meanwhile, differential equation models [6], [10] provide precise, quantitative predictions about the evolution of systems over time, but they rely on extensive parameterization. However, computational advancements, particularly artificial intelligence (AI) and machine learning (ML), are crucial for overcoming these challenges. AI/ML approaches trained on high-content pooled screens are increasingly feasible and cost-effective, providing a foundation for robust computational models [1]. AI based methods are also pivotal for analyzing perturbation proteomics data [11]. Although many tools have been developed to study the response genetic intervention, tools for assessing the responses to pharmacologic compounds and dose responses at the single-cell single-cell level are limited. Therefore, it is critical to perform perturbation modeling at the single-cell level to evaluate the effect of the specific drug concentrations such as benchmark doses as used in toxicology or to model treatment resistance. Therefore, large perturbation datasets and systematic repositories for biological responses to treatment, such as Connectivity Map [12], are indispensable sources that can be used by transfer learning or deep generative models to identify drug response at the single-cell level [13], [14].

In this review, we outline the core principles underpinning perturbation analysis and discuss the methodologies and analytical frameworks, especially AI/ML, used to decode drug and genetic perturbation responses, complex multicellular interactions, and network dynamics We also provide an overview of the current tools and software used for various applications.

## 2 Principles of perturbation modeling

**Fig. 1:**
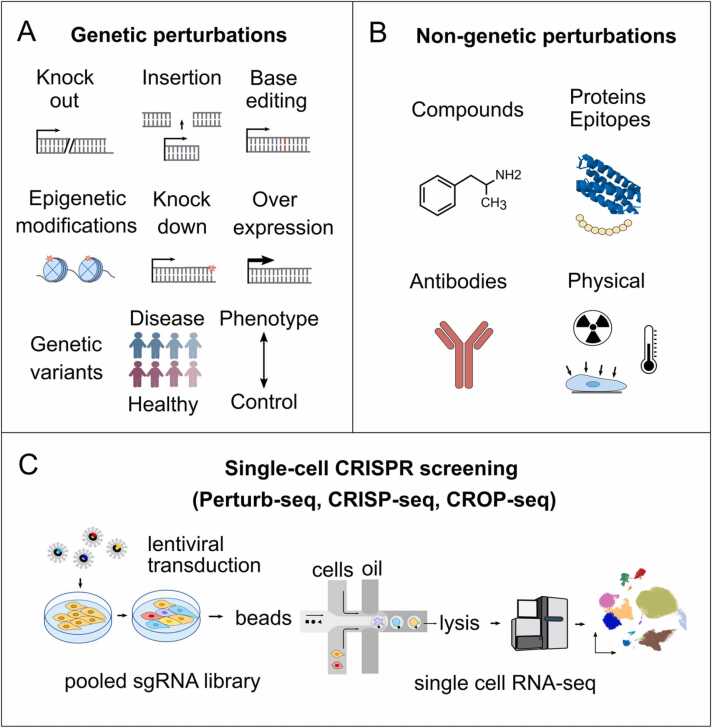
Genetic (A), nongenetic perturbations (B), and methods for genetic manipulation (CRIPSR/Cas9, RNAi) and scRNA-seq read out (C).

When a perturbation, such as a genetic alteration, pharmacological treatment, or environmental stressor, is introduced, cellular homeostasis is disrupted, leading to cascades of molecular changes. Perturbations can be broadly categorized into intrinsic and extrinsic types (Fig. 1). Intrinsic perturbations, such as genetic mutations, gene deletions, and transgene insertions originate within the organism. By contrast, extrinsic perturbations arise from external influences, such as exposure to drugs, cytokine treatments or co-culture conditions replicating the tumor microenvironment [15]. The primary goal of perturbation analysis is mapping these molecular changes to understand their effects on cellular function, signaling pathways, and phenotypic outcomes. Previously, four main objectives in single-cell perturbation modeling were described [2], [15]. These objectives are defined as solvable tasks, each paired with specific evaluation metrics that can be used to assess model performance and guide research in the field [15]. First, a key goal is predicting the responses of cells to various perturbations, including novel changes in gene, protein, and metabolite levels, which can help identify new cell states and markers of drug sensitivity, as evaluated using regression between predicted and observed responses. Second, understanding a compound’s mode of action (MoA), including its molecular targets and underlying biological mechanisms, is crucial for identifying novel compounds or repurposing them in drug discovery. To evaluate the model predictions of affected targets and pathways, classification metrics such as precision and recall can be used. Third, modeling the interactions between genetic and chemical perturbations is essential for developing effective combination therapies. The synergy of different compounds can be estimated using categorical or continuous based multivariate modeling. Lastly, perturbation modeling also extends to predicting chemical structures and generating new small molecules with different biological effects. Similarity scores or classification metrics are used to compare predicted compounds and evaluate their structural features. Together, these objectives provide a comprehensive framework for advancing single-cell perturbation studies and drug discovery.

**Fig. 2:**
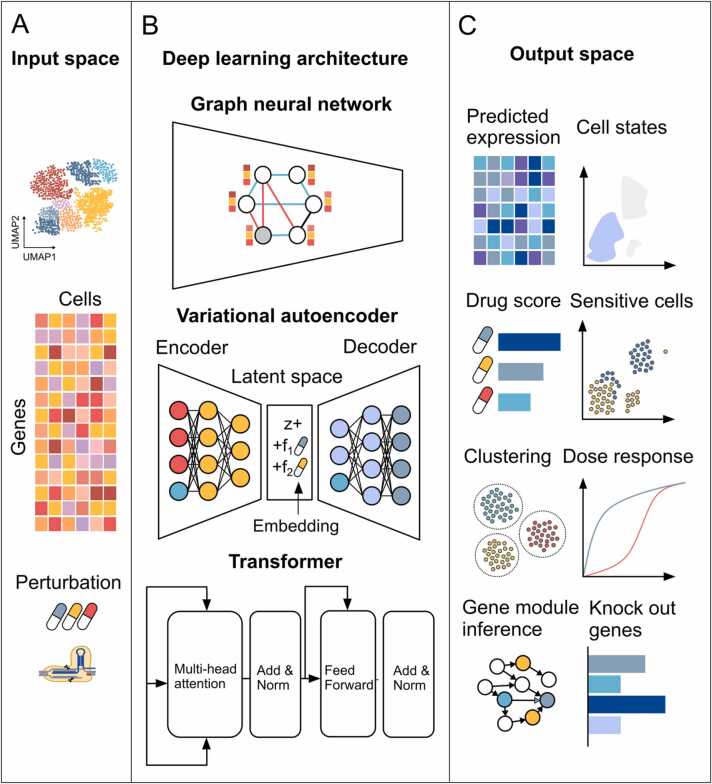
Overview of ML and network models for scRNA-seq perturbation analysis. The schematic illustrates input space (A) various architectures, including graphical neural networks, variational autoencoder, and transformer (B), and output space (C).

A critical aspect of perturbation analysis is understanding the reorganization of molecular networks in response to stimuli [15]. Numerous techniques and models have been developed to analyze perturbation data, addressing the complexity of biological systems (Fig. 2). The most common methods employed in single-cell perturbation analysis are classical statistical and ML inference models, variational autoencoders (VAEs), graphical models, and transformer architectures often applied in foundation models [2], [16] (Table 1).

**Table 1:** Computational and experimental tools for perturbation analyses.

**Tool name**	**Ref.**	**Input data**	**Model/method**	**Description**
**Drug response**				
ASGARD	[82]	scRNA-seq	Predictive pipeline	Matches diseased clusters with normal clusters to score drugs that reverse disease-related expression.
Beyondcell	[83]	scRNA-seq	Therapeutic clustering	Identifies tumor subpopulations with unique drug responses.
CaDRReS-Sc	[84]	scRNA-seq	Recommender systems	Estimates IC50s for cell clusters from gene expression data.
DREEP	[58]	scRNA-seq	Functional enrichment analysis	Predicts drug sensitivity from scRNA-seq data using pharmacogenomic screens, validated across cancer datasets
DrugCell	[85]	Tumor cell mutation and chemical data	Dual-branch deep learning	Models of mutation impact on pathways to predict drug sensitivity.
PRnet	[13]	Bulk and scRNA-seq	Perturbation-conditioned generative model	Integrates bulk and single-cell data via harmonization and transfer learning to predict single-cell drug responses and resistance mechanisms.
scDEAL	[14]	Bulk and scRNA-seq	Deep transfer learning	Uses bulk data to predict single-cell drug response, highlighting key genes.
scDR	[64]	scRNA-seq	Drug response scoring	Predicts drug response from scRNA-seq data and identifies resistance mechanisms.
scDrug	[90]	scRNA-seq	Bioinformatic workflow	Annotates tumor clusters, identifies DEGs, and predicts subpopulation drug response.
**Single-cell analyses**				
scBERT	[34]	scRNA-seq	Foundation model	Learns gene–gene interactions from large-scale scRNA-seq data for accurate cell type annotation and batch effect correction
scGen	[27]	scRNA-seq	VAEs	Combines VAEs and latent space vectors for high-dimensional single-cell gene expression data to accurately model perturbation.
scGPT	[35]	Single-cell multiomics	Foundation model	Offers flexible pipelines for multiomic integration, batch correction, cell type annotation, perturbation prediction, and GRN inference.
scFoundation	[33]	scRNA-seq	Foundation model	Provides several analyses, including gene expression enhancement, tissue and single-cell perturbation prediction, including drug response, cell type annotation and gene module inference.
scMAGeCK	[91]	scCRISPR screens	Robust rank aggregation	Identifies up- and downregulated genes upon gene knockout and constructs a genotype-phenotype network.
scDist	[17]	scRNA-seq	Mixed-effects model	Detects perturbed cell types while reducing false positives from individual and cohort variability.
Augur	[19]	scRNA-seq, STARmap, scATAC-seq	RF classifier	Prioritizes cell types based on their molecular response to a biological perturbation.
PerturbNet	[92]	scCRISPR screens	Deep generative model	Predicts the distribution of cell states induced by unseen chemical or genetic perturbations.
CPA	[52]	Single-cell and bulk RNA-Seq	AE	Prediction of combinatorial perturbation screens for drugs and genes
cycleCDR	[53]	Bulk RNA-seq, bulk proteomics, scRNA-seq	AE	Prediction of cellular responses to novel perturbations (e.g. unseen drugs)
CellOT	[54]	scRNA-seq	Optimal transport	Fully parameterized transport map to predict the cell state. Assumes that the perturbation induces subtle changes.
CellOracle	[22]	scRNA-seq	GRN modeling	Dissecting cell identity via network inference and in silico gene perturbation
MUSIC	[51]	CROP-seq, Perturb-Seq, CRISP-Seq	Topic modeling	Quantitative assessment of the perturbation impact on each gene
MIMOSCA	[3]	scRNA-seq	Perturbation analysis	Designs, performs, and analyzes perturbation scRNA-seq experiments to decode biological dynamics.
GEARS	[23]	scRNA-seq	Deep learning and knowledge graph	Predicts transcriptional responses to single and multigene perturbations using gene-gene relationships
trVAE	[29]	scRNA-seq	Transfer VAE	Predicts gene expression changes after perturbations through learning cell type-specific responses and improving out-of-distribution generalization
**Spatial omics**				
CELLama	[71]	scRNA-seq, ST	Language and the foundation model	Embeds multimodal data for applications such as cell typing and spatial context analysis.
Celcomen	[75]	scRNA-seq, ST	Generative graph neural network	Disentangles intra- and inter-cellular gene regulation to model perturbation effects in spatial and single-cell data
COMMOT	[69]	ST	Collective optimal transport	Infers cell-cell communication by considering ligand-receptor competition and spatial distances.
FlowSig	[64]	Single-cell transcriptomics or ST	Graphical causal modeling	Infers directed intercellular flows, from the input to the intracellular gene expression modules to the output of intercellular signals.
LIANA+	[68]	Single-cell and spatial omics	Multiview learning	Identifies inter- and intracellular signaling and CCC
MISTy	[62]	Multiplexed spatial data	Multiview learning	Extracts spatial omics relationships, such as GRNs and pathway activities.
SCING	[24]	Single-cell transcriptomics or ST	Gradient boosting and mutual information	Infers gene regulatory networks for physiology and disease insights.
SiGra	[70]	ST	Graph representation and the hybrid graph transformer	Enhances ST data to help uncover spatial architecture.
**Experimental methods**				
CROP-seq	[43]	Single-cell transcriptomics	Pooled CRISPR screens and droplet sequencing	Links gRNA expression to transcriptomic responses.
CRISP-seq	[44]	Single-cell transcriptomics	Pooled CRISPR screens	Combines CRISPR-pooled screens with scRNA-seq.
CUT&Tag	[40]	Chromatin profiling	Tagged transposase antibody	Examines histone modifications and transcription factors at single-cell resolution.
ECCITE-Seq	[46]	Single-cell transcriptomics and proteomics	Multimodal CRISPR screen	Captures multiomic data, integrating RNA and protein expression.
MIX-seq	[86]	scRNA-Seq	Multiplexed transcriptomics	Profiles perturbation responses across > 100 cell lines.
Perturb-CITE-Seq	[45]	Single-cell transcriptomics and proteomics	Pooled CRISPR screens	Integrates Perturb-Seq with proteomics using an antibody panel.
Perturb-Map	[63]	Multiplex imaging and ST	CRISPR with Pro-Codes	Analyzes gene knockout effects on tumor structure and immune infiltration.
Perturb-Seq	[3]	Single-cell transcriptomics	Pooled CRISPR screens	Assesses transcription factor effects using perturbation and scRNA-Seq.
sci-CAR	[41]	scRNA-seq and chromatin profiling	Single-cell combinatorial indexing with split-pool barcoding	Integrates RNA expression and chromatin accessibility in single cells.
scATAC-seq	[87]	Single-cell chromatin accessibility	Tn5 transposase tagging	Maps variations in regulatory element accessibility.
scBS-seq	[88]	Single-cell bisulfite sequencing	Bisulfite conversion	Measures DNA methylation in heterogeneous populations.
scTrio-seq	[38], [39]	Single-cell multiomics	RNA release and nuclei sequencing	Analyzes the transcriptome, methylome, and CNVs in the same cells.
TAP-seq	[89]	Single-cell transcriptomics	Genome-scale CRISPR screening	Maps enhancer-target gene interactions.

Classical statistical and ML methods include linear regression, generalized linear models, random forests (RFs), and support vector machines. These methods have been widely used because of their interpretability, and they serve as important baselines for more complex models. For example, scDist uses a linear mixed-effects model to detect transcriptomic differences between conditions, accounting for individual and technical variability to identify cell-specific perturbation responses [17]. Augur uses an RF classifier to rank cell types based on their perturbation sensitivity, calculating the cross-validated area under the receiver operating characteristic curve to distinguish conditions [18]. MIMOSCA integrates linear regression models with interaction terms to analyze perturbation effects while controlling for covariates and minimizing confounding effects [3]. However, classical models struggle to capture non-linear relationships, cell-cell dependencies, and dynamic cell-state transitions [19], [20]. These limitations make advanced architectures such as VAEs, graphical neural networks, and transformers essential for single-cell perturbation analysis.

Gene-regulatory networks (GRNs) can be combined with graphical neural networks to build the foundation for interpretable approaches that represent gene expression regulation as networks or graphs, incorporating components such as transcription factors, splicing factors, noncoding RNAs, and metabolites [21]. GRNs are crucial for perturbation modeling because they capture gene-gene interactions and help predict changes in gene expression during perturbations [2], [16]. For example, CellOracle constructs GRNs by integrating single-cell RNA-seq and ATAC-seq data to model transcriptional connections and permits the study of transcription factor-driven cell fate transitions [22]. Graph-enhanced gene activation and repression simulator uses unperturbed single-cell gene expression data and a specified perturbation set to predict transcriptional responses to gene perturbations [23]. Single cell integrative GRN inference constructs cell type-specific GRNs from scRNA-seq and spatial transcriptomics data by employing gradient boosting regression to infer regulatory relationships [24]. Importantly, these methods both predict cellular responses to perturbations and make them interpretable.

Dimensionality reduction is essential for analyzing high-dimensional single-cell data. Principal component analysis is often used for this purpose, but has limitations in correctly representing global and local structures in scRNA-seq data [25]. Autoencoders (AEs) reduce dimensionality by encoding data into a lower dimensional space and reconstructing it, however, they often overfit, limiting generalizability [26], [27]. VAEs extend AE by introducing a distribution to the latent space [28]. This enables the model to learn representations of the input data. This generalizability allows VAEs to capture both condition- and cell type-specific information [27]. For instance, scGen applies a VAE framework to model single-cell perturbation responses across cell types, species, and experimental conditions [27]. This approach encodes unperturbed and perturbed single-cell gene expression into a latent space. A perturbation vector, representing the difference between the unperturbed and perturbed states, is calculated in this space. The decoder then reconstructs the corresponding gene expression profile, enabling scGen to predict perturbation responses for unseen conditions, such as dose-response effects and infection outcomes [27]. Meanwhile, transfer variational autoencoder extends the VAE framework to predict single-cell perturbation responses while addressing batch effects and domain adaptation challenges [29]. Similarly, the compositional perturbation autoencoder (CPA) builds on the VAE framework by modeling perturbation- and cell type-specific factors separately in the latent space [30]. CPA combines these factors to predict transcriptional responses to unseen drug combinations, doses, or genetic interactions.

Foundation models based on transformer architectures have revolutionized scRNA-seq analysis through pretraining on diverse datasets to create scalable, generalized models [31], [32]. These models capture complex transcriptional patterns, address batch effects, and integrate multiomics data [31], [32]. By applying self-attention mechanisms and multihead attention layers, the transformer models capture dependencies across genes and cells. This ability to integrate information from thousands of genes makes these models exceptionally powerful tools for uncovering gene regulatory networks, cell-cell communication pathways, and cellular responses to perturbations (Fig. 2). Several foundation or large language models have recently been developed for single-cell analysis. Single-cell generative pretrained transformer (scGPT) addresses the challenges of pretraining on large-scale single-cell omics data using a transformer-based architecture trained on more than 33 million cells from 441 studies [33]. The framework has two stages: pretraining on large cell atlases to create general representations, then fine-tuning on smaller datasets for specific tasks [33]. scGPT uncovers cell type- and perturbation-specific biology while generalizing across studies and conditions. scFoundation employs an advanced encoder-decoder architecture with an asymmetric design, that was pretrained on more than 50 million cells spanning more than 100 tissues, cell types, and conditions. This model can be applied for half-maximal effective concentration (IC50) prediction, drug response modeling, and perturbation analysis [31]. Single-cell bidirectional encoder representations from transformers (scBERT) combines the BERT model architecture with gene embeddings to capture transcriptional patterns and gene-gene interactions [34]. Pretrained on large scRNA-seq datasets, scBERT specializes in cell type annotation, perturbation response prediction, and batch correction. Its self-attention mechanism enables the accurate identification of novel cell types and cross-cohort cell type assignments [34]. These foundation models provide scalable frameworks for advancing single-cell analysis, enabling accurate predictions of gene expression, perturbation responses, and cellular behavior across diverse conditions and datasets.

In summary, a range of analytical frameworks from classical models to advanced deep learning techniques have significantly improved our understanding of molecular responses to perturbations. Each approach has its strengths, and combining these methods provides a more comprehensive overview of cellular behavior.

## 3 Perturbation analysis of single-cell data

scRNA-seq has revolutionized biological research, offering an unprecedented level of detail in analyzing cellular responses to perturbations [35]. Despite the growing availability of single-cell omics datasets and resources such as the Human Cell Atlas [36], relatively few single-cell perturbational screens have been developed [15]. scRNA-seq enables researchers to understand the responses of cells to various stimuli with exceptional granularity. Its integration with other modalities, including single-cell proteomics and metabolomics, could expand its capacity to analyze complex cellular responses comprehensively. Combining scRNA-seq with genomic sequencing has provided a detailed understanding of the influence of copy number variations (CNVs) on gene expression at the single-cell level [37]. Furthermore, the integration of single-cell multiomics techniques has facilitated the exploration of epigenetic modifications with high resolution. Techniques such as scTrio-seq [38], [39], single-cell assay for transposase-accessible chromatin sequencing (scATAC-seq), single-cell bisulfite sequencing (scBS-seq), CUT&Tag [40], and sci-CAR [41] offer comprehensive insights into DNA methylation, histone modifications, and chromatin accessibility (Table 1). These advancements have provided a holistic view of the regulatory mechanisms driving cellular behavior, especially under perturbational conditions.

The most promising advancement in the field of genetic perturbation is single-cell CRISPR (scCRISPR) screening. This technique combines CRISPR-mediated gene editing with scRNA-seq to elucidate gene function, identify gene signatures, and evaluate cell states under diverse perturbational scenarios [42]. Perturb-Seq [3], CROP-seq [43] and CRISP-Seq [44] have enhanced scCRISPR screening (Fig. 1C). With guidance by CROP-seq in particular, RNA expression can be directly linked to transcriptome responses. Extensions such as Perturb-CITE-Seq [45] and ECCITE-Seq [46] incorporate proteomic data, providing additional layers of information about the impact of perturbations on both transcriptomic and proteomic levels by using selected antibodies to investigate proteins alongside gene expression data [47].

To support the analysis of scCRISPR screening data, advanced computational tools have been developed. For instance, the tool model-based understanding of single-cell CRISPR screening (MUSIC) [48] applies topic modeling to distinguish cellular perturbation states and identify critical regulatory changes. For each cell, the expression of each gene is analyzed and grouped into a number of topics required to distinguish the different perturbation states. Other computational models, such as CPA [30] and cycleCDR [49], have been designed to predict cellular responses to drug-induced or genetic perturbation. CPA leverages AE-based frameworks for this purpose, and they can handle experiments with multiple gene knockouts, thereby permitting the investigation of interactions between different genes and their combined effects on the cell. Meanwhile, cycleCDR applies two AEs to build a linear model for dynamic cellular behaviors. CellOT [50] uses the optimal transport theory to map subtle transitions between homeostatic and perturbed states, although its performance decreases with highly complex or strong perturbational effects. To improve the training of these models, several implementations have been developed to integrate perturbation data. For example, PerturBase [47] offers scRNA-seq and scATAC-seq data from 122 datasets (approximately 5 million cells) and provides tools to analyze genetic and chemical perturbations. PerturbDB [51] provides 66 Perturb-Seq datasets, featuring transcriptomes from 4.5 million cells and CRISPR-mediated knockdown for cancer research. scPerturb [52] harmonized 44 single-cell datasets for method benchmarking (Supplementary Table 1).

Despite the progress, significant challenges remain. Some computational tools might be less effective for highly complex perturbational responses, indicating a need for continued refinement. As the field advances, the combination of screening data with new multimodal models will improve our understanding of cellular mechanisms.

## 4 Dose-response analysis in single-cell and multiomics contexts

Single-cell methods have become powerful tools for high-throughput drug screening, enabling the prioritization of therapeutic options and the identification of potential drug synergies or resistance clusters within tumors. A key aspect of dose–response analysis is the estimation of the biological activity of a drug, often measured as the IC50, which represents the drug concentration required to reduce a given cellular response (e.g., growth, viability, or gene expression changes) by 50 % [53]. The approaches that model the effects of a drug at the single-cell level can capture these effective doses, key dose-dependent transcriptional responses and pathway perturbations [42], [54].

By distinguishing sensitive and resistant cell populations, scRNA-seq enables the identification of the mechanisms underlying drug resistance and helps refine therapeutic strategies. This makes scRNA-seq ideal for identifying a drug’s MoA, the causal biochemical and molecular pathways linking drug exposure to biological outcomes [42], [55].

Based on their methodological approach, tools for dose-response analyses can be divided into bulk-to-single-cell transfer learning methods, which infer single-cell responses using bulk RNA-seq data, and direct single-cell prediction methods, which train models directly on single-cell data. Response modeling can be performed at the cluster or single-cell level. Bulk-to-single-cell transfer learning approaches use bulk RNA-seq drug-response datasets to train models that can then be applied to scRNA-seq data. This method relies on established bulk databases, such as Genomics of Drug Sensitivity in Cancer (GDSC) [56] and Cancer Cell Line Encyclopedia (CCLE) [57], to train the models that require large amounts of data for the training process. Examples of tools employing this strategy include single-cell drug efficacy prediction with adversarial learning (scDEAL) [14], a deep transfer learning framework designed to predict drug responses at the single-cell level by integrating bulk and scRNA-seq data [14]. It employs two denoising AEs to extract low-dimensional gene features separately from bulk and scRNA-seq data [14]. Then, these representations are aligned through a domain-adaptive neural network. This approach ensures that drug response labels learned from bulk datasets can be effectively transferred to single-cell resolution. In addition, it applies integrated gradient interpretation to identify key gene signatures contributing to drug sensitivity or resistance [14]. Another approach to modeling drug responses is through perturbation-conditioned deep generative models, which learn transcriptional changes directly from perturbation data. PRnet is a deep generative model designed to predict gene expression responses to novel chemical perturbations at both the bulk and single-cell levels [13]. It integrates unperturbed transcriptional profiles and chemical perturbation data, and map them into a latent space that models how compounds influence gene activity [13]. The framework includes three core components: Perturb-adapter, which encodes chemical structures using SMILES representations, Perturb-encoder, which processes perturbation effects on baseline gene expression, and Perturb-decoder, which predicts transcriptional changes [13]. Another bioinformatics approach named drug response estimation from single-cell expression profiles (DREEP) is designed to predict drug sensitivity or resistance from scRNASeq data [58]. It integrates pharmacogenomic datasets, including GDSC, CTRP, and PRISM, to construct genomic profiles of drug sensitivity (GPDS)-ranked gene lists that capture associations between gene expression and drug potency [58]. Using gene set enrichment analysis (GSEA), DREEP compares single-cell expression profiles against GPDS signatures to the estimate drug response. A positive enrichment score indicates resistance-associated gene expression, whereas a negative score suggests sensitivity [58].

Unlike bulk-to-single-cell approaches, direct single-cell prediction methods operate exclusively on scRNA-seq data, capturing cell-specific responses and providing insights into cellular heterogeneity. For instance, scDR [59] predicts gene expression changes and drug-response scores to distinguish resistant and sensitive cell types across cancer types. Additional tools such as scDRUG [90] and Beyondcell [83] aggregate data from cell populations to reduce noise and improve computational efficiency. The most comprehensive repositories for drug responses are PRISM, CCLE, and GDSC [56], [57], [60], which provide extensive information on drug sensitivity and gene expression in cell lines mostly related to cancer. Despite newer developments such as PerturBase [47] and CeDR Atlas [61], which compile single-cell drug response data, no truly comprehensive resource that captures all necessary aspects for in-depth modeling exists. A unified, extensive database that covers a wide range of cell types, perturbation conditions, and dose-response relationships is missing. Supplementary Table 1 summarizes some of the largest currently available single-cell drug response studies, but more effort is needed to develop an all-encompassing resource that can support more reliable predictions and cross-study analyses.

Overall, single-cell methods enable high-throughput drug screening and detailed exploration of drug MoA. The continued development of these tools and resources will advance our understanding of therapeutic heterogeneity, identify resistance mechanisms, and uncover novel therapeutic targets in oncology research.

## 5 Spatial approaches for perturbation modeling

scRNA-seq data has several advantages in perturbation analyses, including the possibility of deciphering different cell types and stages. However, spatial contexts, which are critical for understanding tissue architecture and cell-cell interactions, are lost [62]. Spatial transcriptomics (ST) addresses this limitation by preserving spatial information while profiling gene expression. Although ST has primarily been used to analyze tissue organization, it is increasingly being adapted to investigate cellular responses to perturbations. One example is Perturb-Map, which employs protein barcoding (Pro-Codes) to mark cells expressing CRISPR gRNAs. These Pro-Codes can be used to assess the effects of gene knockout on tumor architecture and immune cell infiltration through multiplex imaging and ST [63]. However, the application of ST to perturbational studies remains limited, partly because of the lack of paired ST data before and after perturbation [64]. Consequently, tools that are not specifically designed for perturbation screens but can be adapted to compare control and perturbed states in different scenarios have been applied (Table 1). One of the advantages of ST in perturbation biology is hypothesis generation. The large datasets produced by ST permit the identification of novel ligand-pathway interactions, as demonstrated by tools such as multiview intercellular spatial modeling (MISTy). MISTy uses explainable ML on multiplexed spatial technologies, such as ST and imaging mass cytometry, to understand marker interactions by profiling intra- and intercellular relationships. It constructs models to describe different spatial contexts, including the intrinsic, local, and tissue views [62]. Other tools such as PROGENy [65] and OmniPath [66] can be included to add information about signaling pathway activity and ligand gene expression, respectively [62]. By integrating such tools, researchers can better understand the influence of perturbations on signaling pathways and cellular communication. Furthermore, perturbation can alter cell-cell communication (CCC), which can be analyzed using transcriptomic data by extracting information from receptor-ligand interaction databases such as CellPhoneDB [67]. LIANA+ extends MISTy’s multiview approach, enabling the joint modeling of combinations of complex tissue structures and functions. It can infer receptor-ligand interactions from ST data in an unsupervised manner or from scRNA-seq data across multiple conditions [68]. Methods such as communication analysis by optimal transport (COMMOT) extend this approach by modeling the directionality of CCC in ST data, which is essential for understanding the dynamic signaling response to perturbations. This is made possible by using collective optimal transport to infer CCC in space by introducing the distributions of ligands and receptors and enforcing spatial constraints [69]. However, a limitation of transcriptomics-based CCC inference is the lack of direct protein-level data, leading to potential false positives [69]. In addition, FlowSig allows the inference of intercellular flows using ST or scRNA-seq data. It employs causal modeling to construct directed graphs and identify signal flow and is sufficient to map inflowing intercellular signals (inputs) to gene expression modules that mediate intracellular responses and generate outflowing intercellular signals (outputs) at each spatial location [64]. These graph-based approaches are intuitive for ST, as the spatial relationships between cells can be naturally represented [70]. They can also help to improve the analysis of ST data, as demonstrated by single-cell spatial elucidation through image-augmented graph transformer (SiGra), because they usually have a low total number of transcripts per cell, noisy data and significant zeros. SiGra enhances gene expression data by combining single-cell and spatial multimodal data, including imaging and transcriptomics, into a graphical model with three graph transformer-based encoder-decoders. This enhanced gene expression facilitates insights into CCC and other biological discoveries [55]. Both spatial and single-cell information can be captured through transcriptomics, as well as genomics, epigenomics, proteomics, and metabolomics, providing a more comprehensive picture of the effects of perturbations on cellular systems. However, these different types of data must be integrated to obtain information about perturbational changes. Emerging ML frameworks, such as foundation models, can integrate different types of data modalities. For example, cell embedding leverage language model abilities (CELLama) embeds multimodal data into a shared space, enabling the integration of scRNA-seq, ST data, and metadata for enhanced predictions [71]. SCING can also integrate multiomics data, including ST data and protein expression data from immunohistochemistry. Using scRNA-seq or ST, SCING infers GRNs, which are crucial for perturbation analysis, as they reveal changes in gene-gene interactions [24]. Despite these advances, ST and scRNA-seq face barriers to clinical translation, including high costs and technical complexity. To address this, tools have emerged to predict gene expression from the frequently used imaging technique of hematoxylin and eosin (H&E) staining (e.g., HE2RNA, Hist2ST [72], [73]). Furthermore, tools such as tumor edge structure and lymphocyte multi-level annotation (TESLA) can use H&E stainings to enhance the spatial resolution of ST for the tumor region annotation [74]. In the future, such techniques could be used in clinical contexts to infer perturbation effects at the tissue level.

In summary, although currently available tools for spatial analyses are not explicitly designed for perturbation studies, they can be adapted to address perturbation-related questions, including hypothesis generation or CCC inference. One recent approach named Celcomen [75] focused on spatial causal disentanglement for single-cell and tissue perturbation modeling through a generative graphical neural network. It is anticipated that novel computational tools will be developed to specifically address the integration challenges associated with perturbation biology.

## 6 Applications

**Fig. 3:**
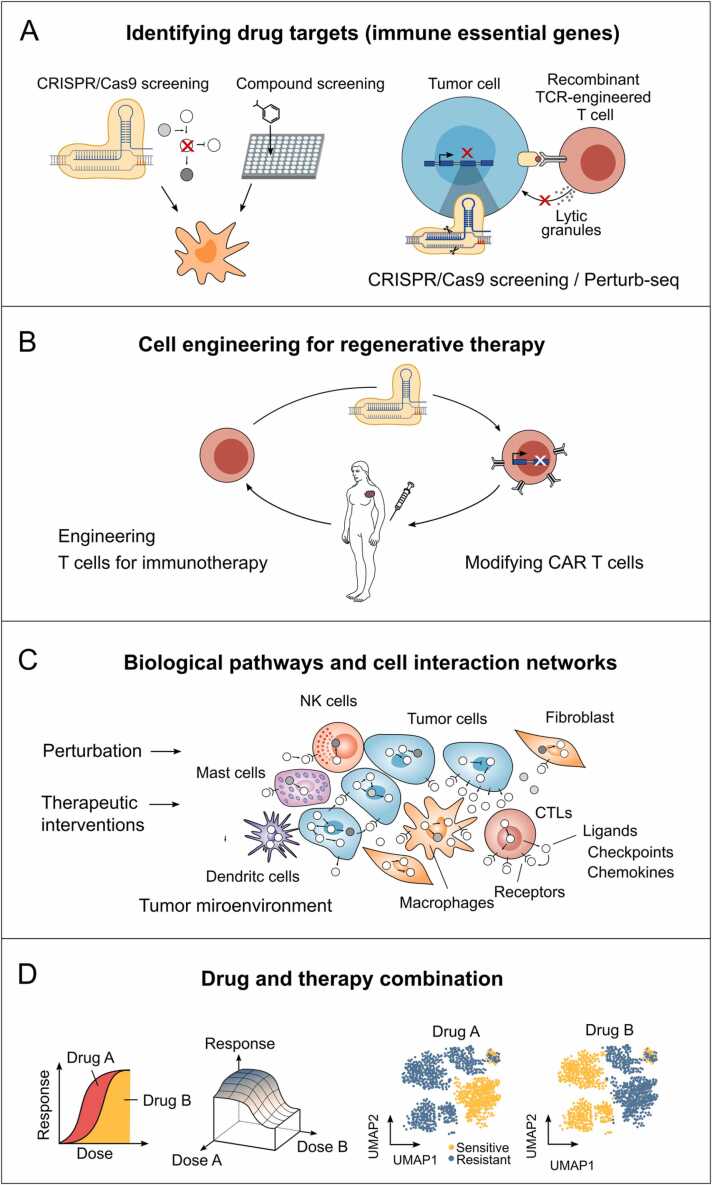
Applications for perturbation modeling and screening.

Technologies such as CRISPR/Cas9 and RNAi can be used to introduce various genetic manipulations that lead to changes in the function or expression of certain genes. These methods are crucial in screening for genetic modifications that result in specific phenotypic or functional changes in cells, potentially facilitating the identification of drug targets. For instance, screening of cancer cell lines can facilitate the development of targeted therapies. Moreover, these techniques are highly valuable for immunotherapy. By using a two-cell co-culture model that systematically modifies genes in tumor cells and reads out the cytotoxic function of T cells, researchers can identify immune-essential genes (Fig. 3A). This could even be improved when combined with scRNA-seq techniques, such as Perturb-Seq or CROP-seq to enable large-scale perturbation studies with transcriptional readouts.

Another promising application relevant to immunotherapy and regenerative cell therapy is to identify the optimal manipulation for engineered T cells (or CAR T cells, Fig. 3B). In general, pooled genetic perturbation screens are extremely powerful, and they can be applied in many applications, allowing the comprehensive characterization of cell models such as organoids. Another emerging technology is optical pooled screens, which use imaging or in situ sequencing or hybridization as readouts [1]. In addition, spatially resolved transcriptomics provides a detailed view of gene expression in tissue contexts. Although challenges such as sensitivity limitations and labor-intensive protocols remain [76], with recent developments involving the use of RNA imaging-based techniques (MERFISH) or the padlock-based Xenium in situ platform (10X), a readout at the single cell resolution of thousands of genes has become possible. This expands the scope of our analyses making it possible to investigate the interaction between the same cell types, but also between different cell types, and how they can be affected by certain perturbations or treatments. The tumor microenvironment in solid tumors represents an ideal application, in which the communication and co-localization between tumor cells and a number of other cell types such as cytotoxic T lymphocytes and changes in their patterns by certain interventions can be investigated (Fig. 3C). New computational tools, such as the spatial foundation model CELLama [71], are being developed to integrate spatial data with single-cell information aiming to enhance spatial context analysis and perturbation prediction. Interestingly, a spatial CRISPR screen (PerturbMap) show its ability to identify tumor specific modulation of macrophages in the tumor microenvironment in ovarian cancer [77]

From cancer treatment studies, an increasing number of scRNA-seq data, as outlined in Supplementary Table 1, are available. If systematically acquired, analyzed, and integrated into perturbation atlases, these datasets could contribute to our understanding of resistance mechanisms. The availability of other data modalities is limited; therefore, it is difficult to address whether the integration of other readouts can improve characterization of drug responses [15]. Perturbation modeling could also be used to investigate drug and therapy combinations and their responses. One possibility to expand genetic screens for target identification is analyzing synthetic lethal interactions using simultaneous perturbations of two genes resulting in cell death or combining gene perturbation with small molecules or other compounds that can be used to elucidate the MoAs of drugs. In any case, the response to drug combinations can also be investigated directly by interaction modeling. For multicellular systems additional readouts from scRNA-seq are extremely valuable for identifying mutually resistant cell populations and prioritizing drug combinations (Fig. 3D). Single-cell analyses would also be relevant in investigating drug repurposing and dose-response analyses to determine effective concentrations and toxicologic thresholds. However, few single-cell datasets are available considering the response at different concentrations.

## 7 Conclusions and outlook

The promise of foundation models including scFoundation [31] lies in their ability to enhance our understanding of cellular and tissue-level biology. However, their progress requires continuous innovation in the computational approaches and even more importantly huge amounts of training data. The development of comprehensive resources such as single cell atlases including tumor tissues [78], [79] has also sparked the generation of perturbation cell and tissue atlases [1]. PerturbAtlas and PerturbDB represent such platforms, which systematically map gene functions and the effects of genetic perturbations across various cell types, accelerating discoveries, and providing benchmarks for model evaluation. Specifically for tissue-level studies, spatial approaches such as PerturbMap are promising, but there is a lack of paired spatial transcriptomics data before and after perturbation, so the number of approaches for perturbation analysis in spatial settings remains limited. With increasing availability of ST data, further computational approaches will be adapted or new ones developed to reveal changes in the spatial cell distribution, which will ultimately improve the situation for perturbation modeling in this context.

Benchmarking efforts such as those outlined in Therapeutics Data Common, which provides ML datasets for drug discovery and development [80] should prompts efforts for standardized and accessible data or tasks. We anticipate a growing number of ML models for perturbation analyses with similar impact as we observed for protein structure predictions [81].In conclusion, recent developments in AI/ML as well as emerging single cell technologies hold great promise for perturbation modeling to improve drug development and personalized medicine. However, continued collaboration between experimental and computational biologists will be critical to overcome current limitations and develop tools that are both predictive and interpretable. The combination of foundation models and perturbation atlases offers immense potential for advancing our understanding of cellular behavior and disease mechanisms.

## CRediT authorship contribution statement

**Monfort-Lanzas Pablo:** Writing – review & editing, Writing – original draft, Visualization, Methodology, Investigation, Conceptualization. **Hackl Hubert:** Writing – review & editing, Writing – original draft, Visualization, Supervision, Project administration, Investigation, Funding acquisition, Conceptualization. **Madersbacher Leonie:** Writing – review & editing, Writing – original draft, Visualization, Methodology, Investigation. **Rungger Katja:** Writing – review & editing, Writing – original draft, Visualization, Methodology, Investigation, Data curation.

## Declaration of Generative AI and AI-assisted technologies in the writing process

During the preparation of this work the authors used DeepL and chatGPT-4.0 in order to improve readability and language. After using this tool/services, the authors reviewed and edited the content as needed and took full responsibility for the content of the publication.

## Declaration of Competing Interest

The authors declare no conflict of interest.

## Appendix A Supplementary material

Supplementary Table 1 Datasets used in the perturbation analyses
